# Mutation-Specific Phenotypes in hiPSC-Derived Cardiomyocytes Carrying Either Myosin-Binding Protein C Or *α*-Tropomyosin Mutation for Hypertrophic Cardiomyopathy

**DOI:** 10.1155/2016/1684792

**Published:** 2015-12-28

**Authors:** Marisa Ojala, Chandra Prajapati, Risto-Pekka Pölönen, Kristiina Rajala, Mari Pekkanen-Mattila, Jyrki Rasku, Kim Larsson, Katriina Aalto-Setälä

**Affiliations:** ^1^BioMediTech, University of Tampere, 33014 Tampere, Finland; ^2^School of Information Sciences, University of Tampere, 33014 Tampere, Finland; ^3^Medical School, University of Tampere, 33014 Tampere, Finland; ^4^Heart Hospital, Tampere University Hospital, 33521 Tampere, Finland

## Abstract

Hypertrophic cardiomyopathy (HCM) is a genetic cardiac disease, which affects the structure of heart muscle tissue. The clinical symptoms include arrhythmias, progressive heart failure, and even sudden cardiac death but the mutation carrier can also be totally asymptomatic. To date, over 1400 mutations have been linked to HCM, mostly in genes encoding for sarcomeric proteins. However, the pathophysiological mechanisms of the disease are still largely unknown. Two founder mutations for HCM in Finland are located in myosin-binding protein C (*MYBPC3-Gln1061X*) and *α*-tropomyosin (*TPM1-Asp175Asn*) genes. We studied the properties of HCM cardiomyocytes (CMs) derived from patient-specific human induced pluripotent stem cells (hiPSCs) carrying either* MYBPC3-Gln1061X* or *TPM1-Asp175Asn* mutation. Both types of HCM-CMs displayed pathological phenotype of HCM but, more importantly, we found differences between CMs carrying either *MYBPC3-Gln1061X* or *TPM1-Asp175Asn* gene mutation in their cellular size, Ca^2+^ handling, and electrophysiological properties, as well as their gene expression profiles. These findings suggest that even though the clinical phenotypes of the patients carrying either *MYBPC3-Gln1061X* or *TPM1-Asp175Asn* gene mutation are similar, the genetic background as well as the functional properties on the cellular level might be different, indicating that the pathophysiological mechanisms behind the two mutations would be divergent as well.

## 1. Introduction

Hypertrophic cardiomyopathy (HCM) is one of the most common genetic cardiac diseases with worldwide prevalence of 1 : 500, as well as the most common cause of sudden cardiac death (SCD) among young competing athletes. HCM is inherited in an autosomal dominant pattern. Nevertheless, a large clinical diversity and age-related penetrance are typical for HCM. On the tissue level, HCM is characterized by the disarray of cardiomyocytes (CMs) and fibrosis of cardiac tissue, as well as thickened interventricular septum or free left ventricular wall. Clinical symptoms include arrhythmias, progressive heart failure, and even SCD, but on the other hand the mutation carrier can be completely asymptomatic. Altogether more than 1400 mutations in 11 genes encoding for the sarcomeric proteins have been identified and related to HCM. The majority of the mutations are found either in the *β*-myosin heavy chain (*MYH7*) or in the myosin-binding protein C (*MYBPC3*) genes [1]. In Finland, two founder mutations located in* MYBPC3* and *α*-*tropomyosin* (*TPM1*) genes and one common mutation in* MYH7* gene together account around 24% of all Finnish HCM cases [2, 3].

Although the genetic information related to HCM has been growing in the recent years due to the development of sequencing technologies, exact information of the disease mechanisms remains unclear. Thus, current medication of the disease is directed toward the symptom relief and there is no specific therapy to prevent the onset or progression of the disease [1]. Most of the HCM studies have been conducted with model systems, mainly either with transgenic mice or by studying human tissues obtained from surgical myectomy from end-stage HCM patients [4]. However, animal models carry only the mutated gene lacking the rest of the genome and myectomy samples are obtained from patients in the late stage of HCM development. Therefore, the discovery of the human induced pluripotent stem cells (hiPSCs) has offered a new valuable tool to model HCM and other cardiac diseases and to study the underlying disease mechanisms [5]. To date, hiPSCs have already been used to model a variety of cardiac diseases: electrical defects, for example, long-QT syndrome [6–8] and catecholaminergic polymorphic ventricular tachycardia (CPVT) [9, 10] as well as cardiomyopathies including dilated cardiomyopathy (DCM) [11] and HCM [12–14].

Here we have derived hiPSCs from patients carrying two of the Finnish HCM founder mutations either in* MYBPC3* (*MYBPC3-Gln1061X*) or in* TPM1* (*TPM1-Asp175Asn*) gene. We have differentiated the patient-specific hiPSCs into CMs and compared the phenotypes of the diseased and control CMs.

## 2. Materials and Methods

### 2.1. Ethical Issues

This study was conducted in accordance with the Ethics Committee of Pirkanmaa Hospital District to establish, culture, and differentiate hiPSC lines (R08070). Skin biopsies for hiPSC establishment were received from the Heart Hospital, Tampere University Hospital, Tampere, Finland. Patients donating skin biopsies signed an informed consent after receiving both oral and written descriptions of the study. The teratoma assay, described in Section 2.3.6, was approved by ELLA-Animal Experiment Board of Regional State Administrative Agency for Southern Finland (ESAVI/6543/04.10.03/2011).

### 2.2. Generation and Culture of Patient-Specific hiPSC Lines

hiPSC lines were generated from skin's fibroblasts either with Sendai reprogramming vectors* OCT4*,* KLF4*,* c-MYC*, and* SOX2* using CytoTune-iPS Reprogramming Kit (Life Technologies Ltd., Paisley, UK) according to the manufacturer's instructions or by using pMX retroviral vectors* OCT4*,* KLF4*,* c-MYC*, and* SOX2* with or without Cre-LoxP site as described earlier [6, 15]. UTA.13602.HCMT, UTA.02912.HCMT, and UTA.04511.WT hiPSC lines were generated by using Sendai vectors and UTA.07801.HCMM and UTA.06108.HCMM by using pMX retroviral vectors with Cre-LoxP site and UTA.04602.WT was generated by using pMX retroviral vectors without Cre-LoxP site. In the present study, one line of each patient was used. hiPSC lines were derived and cultured on mouse embryonic fibroblast (MEF) feeder cell layers (26000 cells/cm^2^, CellSystems Biotechnologie Vertrieb GmbH, Troisdorf, Germany) in human pluripotent stem cell (hPSC) culture medium consisting of knockout-DMEM (ko-DMEM, Gibco, Life Technologies Ltd.) supplemented with 20% knockout serum replacement (ko-SR, Gibco, Life Technologies Ltd.), 1% nonessential amino acids (NEAA, Lonza Group Ltd., Basel, Switzerland), 2 mM GlutaMax (Gibco, Life Technologies Ltd.), 50 U/mL penicillin/streptomycin (Lonza Group Ltd.), 0.1 mM 2-mercaptoethanol (Gibco, Life Technologies Ltd.), and 4 ng/mL basic fibroblast growth factor (bFGF, PeproTech, Rocky Hill, NJ, USA).

### 2.3. Characterization of hiPSC Lines

#### 2.3.1. Mutation Analysis by Genotyping

DNA samples from the hiPSC lines were prepared with TaqMan Sample-to-SNP Kit (Applied Biosystems, Life Technologies Ltd.) and the presence of* MYBPC3-Gln1061X* and* TPM1-Asp175Asn* mutation in the patient-specific hiPSC lines was confirmed by custom TaqMan SNP Genotyping Assays (Applied Biosystems, Life Technologies Ltd.) according to the manufacturer's instructions. In the genotyping assays,* MYBPC3*-gene as well as* TPM1*-gene was amplified with specific primers. Furthermore, the presence of the mutations was assessed with mutation-specific FAM labeled probes. VIC labeled probes were used to assess the presence of the wild type allele. Sequences for the primers and probes used in the assay are listed in Supplementary Table 1 (see Supplementary Material available online at http://dx.doi.org/10.1155/2016/1684792).

#### 2.3.2. The Expression of Mutant and Wild Type Alleles in hiPSC-Derived CMs

RNA samples were collected and extracted from hiPSC-derived CMs (UTA.04511.WT, UTA.02912.HCMT, UTA.07801.HCMM, and UTA.06108.HCMM) with Norgen's Total RNA Purification Plus Kit (Norgen Biotek Corp., Ontario, Canada) according to manufacturer's instructions. 50–100 ng of RNA was transcribed to cDNA by High-Capacity cDNA Reverse Transcription Kit (Applied Biosystems, Life Technologies Ltd.). The expression of* TPM1-Asp175Asn* or* MYBPC3-Gln1061X* mutation on mRNA level in the hiPSC-derived CMs was studied by Custom TaqMan SNP Genotyping Assays (Applied Biosystems, Life Technologies Ltd.) similarly as that for genotyping described above. Sequences for the primers and probes used in the assay are listed in Supplementary Table 1.

#### 2.3.3. Immunocytochemistry

Undifferentiated hiPSC colonies were fixed with 4% paraformaldehyde (PFA, Sigma-Aldrich, Saint Louis, USA), stained with primary antibodies for Nanog (R&D systems Inc., Minneapolis, MN, USA), OCT4 (R&D systems Inc.), SOX2 (Santa Cruz Biotechnology, Santa Cruz, CA, USA), TRA-1-60 (Millipore, Billerica, MA, USA), and TRA-1-81 (Millipore), and visualized with secondary antibodies as described before [16]. Finally, the cells were mounted with Vectashield (Vector Laboratories Inc., Burlingame, CA, USA) containing 40,6-diamidino-2-phenylindole (DAPI) for the nuclei staining and imaged with an Olympus IX51 phase contrast microscope equipped with fluorescence optics and Olympus DP30BW camera (Olympus Corporation, Hamburg, Germany).

#### 2.3.4. RT-PCR

The RNA was extracted from the hiPSC lines by NucleoSpin RNA II Kit (Macherey-Nagel GmbH & Co., Düren, Germany) and 500–1000 ng of RNA was transcribed to cDNA by High-Capacity cDNA Reverse Transcription Kit (Applied Biosystems, Life Technologies Ltd.). The presence of pluripotency genes* Nanog*,* SOX2*,* REX1*,* OCT4*, and* c-MYC* and the absence of virally imported exogenes (*OCT4*,* SOX2*,* c-MYC*, and* KLF4*) were confirmed by RT-PCR.* GAPDH* was used as an endogenous control. The primer sequences for pluripotency genes and virally imported exogenes have been published earlier [5]. The primer sequences used for detection of Sendai transgenes are described in CytoTune-iPS Reprogramming Kit's manual (Life Technologies Ltd.).

#### 2.3.5. Karyotype Analysis

The karyotypes of hiPSC lines were studied by G-banding (Medix Laboratories, Espoo, Finland) or by KaryoLite assay [17] (Turku Centre for Biotechnology, University of Turku, Turku, Finland).

#### 2.3.6. Pluripotency Analysis

The pluripotency of hiPSC lines was confirmed* in vitro* by embryoid body (EB) formation and* in vivo* by teratoma assay. hiPSCs were removed from feeder cell layer and cultured in suspension to form EBs. The EBs were cultured in EB medium consisting of ko-DMEM supplemented with 20% fetal bovine serum (FBS, Biosera, Boussens, France), 1% NEAA (Lonza Group Ltd.), 2 mM GlutaMax (Invitrogen, Life Technologies Ltd.), and 50 U/mL penicillin/streptomycin (Lonza Group Ltd) for 4–6 weeks before RNA extraction. 200 ng of RNA was transcribed to cDNA for the RT-PCR analysis. The presence of all three germ layers, endoderm (*AFP, SOX17*), ectoderm (*SOX1, NESTIN*, and* Musashi*), and mesoderm (*KDR, alpha cardiac actin*), was studied with RT-PCR.

For* in vivo* pluripotency assay, hiPSCs were injected under the testis capsule of nude mice and the formed teratomas were collected and fixed with 4% PFA 8 weeks after the injection. Teratomas were embedded in paraffin, cut in sections, and stained with haematoxylin and eosin.

### 2.4. Differentiation of Cardiomyocytes

hiPSCs were differentiated into CMs by coculturing with mouse visceral endodermal-like cells (END-2) (Hubrecht Institute, Utrecht, Netherlands) as described before [18]. After 15–30 days beating areas were cut from cocultures and dissociated into single cells in EB medium by Collagenase A (Roche Diagnostics, Mannheim, Germany) as described earlier [18] and plated to 0.1% gelatin-coated cover slips or well plates for further analysis.

### 2.5. Characterization of hiPSC-Derived Cardiomyocytes

#### 2.5.1. Immunocytochemistry and Image Analysis

Dissociated CMs were fixed with 4% PFA and stained with Troponin T (cTnT, 1 : 2000, ab64623, Abcam, Cambridge, MA, USA), MYBPC (1 : 400, sc-166081, Santa Cruz Biotechnology), and TPM1 (1 : 200, sc-73225, Santa Cruz Biotechnology) primary antibodies, followed by labeling with secondary antibodies. Images were obtained with Olympus IX51 phase contrast microscope equipped with fluorescence optics and Olympus DP308W camera (Olympus Corporation) or with Zeiss AxioScope A1 fluorescent microscope and Zeiss AxioCam MRc5 camera (Carl Zeiss, Jena, Germany). Size of the Troponin T stained CMs was analyzed from 46 to 50 CMs in each cell line by in-house made software (unpublished method). CMs were analyzed from pictures obtained with Olympus IX51 phase contrast microscope. The proportion of multinucleated CMs was determined from the same images (46–50 CMs/cell line).

#### 2.5.2. Ca^2+^ Imaging

The clusters of CMs were cut, dissociated, plated on 0.1% gelatin-coated coverslips, and cultured for 1, 3, and 6 weeks. To study the Ca^2+^ handling properties of hiPSC-derived CMs, cells were loaded with 4 *μ*M Fura-2 AM (Molecular Probes, Life Technologies Ltd.) for 30 minutes in perfusate medium. The perfusate medium consisted of (in mM) 137 NaCl, 5 KCl, 0.44 KH_2_PO_4_, 20 HEPES, 4.2 NaHCO_3_, 5 D-glucose, 2 CaCl_2_, 1.2 MgCl_2_, and 1 Na-pyruvate dissolved in H_2_O. pH of the perfusate medium was adjusted to 7.4 with NaOH. The coverslip, containing the dissociated hiPSC-derived CMs, was mounted to an RC-25 recording chamber and continuously perfused with perfusate medium preheated to 35-36°C by an SH-27B inline-heater controlled by a TC-324B unit (all from Warner Instruments Inc., Hamden, USA). The perfusion was controlled by a gravity driven VC^3^8 application system (ALA Scientific Instruments Inc., NY, USA). Coverslip was perfused for 15 minutes for Fura-2 AM deesterification before experimental recordings. Ca^2+^ handling of spontaneously beating CMs was imaged with an inverted IX70 microscope using UApo/340 x20 air objective (Olympus Corporation) and ANDOR iXon 885 CCD camera (Andor Technology, Belfast, Northern Ireland) synchronized with a Polychrome V light source by a real time DPS control unit. TILLvisION or Live Acquisition software (TILL Photonics, Munich, Germany) was used for recording. Fura-2 AM was excited at 340 nm and 380 nm light and the emission was recorded for 10–30 seconds at 505 nm.

For Ca^2+^ imaging analysis, single beating CMs were selected as regions of interests and background noise, recorded from a cell-free area in the same coverslip, was subtracted before further processing. Data is presented as ratios of 340/380 nm (F340/F380). The spontaneously beating CMs were divided into five different rhythm categories based on the abnormalities observed in their Ca^2+^signals: normal beating with regular peaks (normal); more than three peaks which do not return to the baseline (oscillation); signals with small or middle sized amplitude events in the beginning, in the end, or in between two Ca^2+^ spikes (low/middle peaks); two or three peaks which do not return to the baseline (double peaks); Ca^2+^ spikes with prolonged rise or decay time (plateau abnormality). In the low/middle peaks category, the small amplitude was at least 10% from the preceding Ca^2+^ spike amplitude. Full-length, 10–30 seconds long recordings were analyzed, while most of the analyzed recordings were 12 seconds long. The distribution of CMs in different categories is presented for each cell lines separately.

#### 2.5.3. Electrophysiological Measurements: Recording and Analysis of Action Potentials

The action potentials (APs) were recorded by conventional patch clamp [19] in perforated patch configuration using Amphotericin B [20] in final concentration of 0.24 mg/mL [8]. Data acquisition was conducted using Axon Series 200B patch-clamp amplifier connected to Digidata 1440a AD/DA converter driven by pCLAMP 10.2 software (all from Molecular devices LLC). On the day of use, the coverslips containing dissociated hiPSC-derived CMs were transferred to RC-24N recording chamber (Warner Instruments Inc.) and mounted on an inverted Olympus IX71 microscope (Olympus Corporation). The patch electrodes had tip resistance of 3.0–3.5 MΩ and contained the following intracellular solution (in mM): 132 KMeSO_4_, 20 KCl, 1 MgCl_2_, and 1 CaCl_2_ (pH was adjusted to 7.2 with KOH). The extracellular solution contained (in mM) 143 NaCl, 4.8 KCl, 1.8 CaCl_2_, 1.2 MgCl_2_, 5 glucose, and 10 HEPES (pH was adjusted to 7.4 with NaOH). The preheated extracellular solution was continuously perfused with similar setup compared to what is presented in Section 2.5.2. Patch pipettes (Harvard Apparatus Ltd., Holliston, MA, USA) were freshly prepared using PC-10 micropipette puller and then flame-polished with MF-830 microforge (both from Narishige Int., Tokyo, Japan).

APs were recorded in the gap-free mode in the current clamp from the spontaneously beating hiPSC-derived CMs. Current-clamp recordings were digitally sampled at 20 kHz and filtered at 2 kHz using low pass Bessel filter on recording amplifier. Beats per minute (BPM), AP duration (APD_50_ and APD_90_), AP amplitude (APA), and maximum diastolic potential (MDP) were analyzed from the recorded APs by using Origin 9.1 (OriginLab Corp., Northampton, USA). Only ventricular-like waveforms are presented here to avoid any biasness among different hiPSC lines. The ventricular-like CMs were characterized by APD_90_/APD_50_ < 1.3 and APA > 90 mV.

#### 2.5.4. Real-Time qRT-PCR Analysis

After one week of culture, dissociated CMs were collected into a lysis solution buffer of CellsDirect One-Step qRT-PCR Kit (Life Technologies Ltd.) according to the manufacturer's instructions. Two replicate samples were collected and stored at −70°C until the DNase I digestion and reverse transcription-specific target amplification (RT-STA) by using CellsDirect One-Step qRT-PCR Kit. Real-Time qPCR was performed with Biomark HD system (Fluidigm Corp., San Francisco, USA) according to the manufacturer's instructions. The TaqMan assays (Life Technologies Ltd.) used in the qRT-PCR are collected in Table 1. All samples were analyzed in duplicate and the fold changes were calculated by the 2^−ΔΔCT^ method [21].* EEF1A1* and* GAPDH* genes were used as endogenous control genes and UTA.04511.WT cell line was used as a calibrator.

#### 2.5.5. Western Blot

hiPSC-derived CMs were lysed in M-PER protein extraction reagent (Thermo Scientific, Life Technologies Ltd.), supplemented with complete protease inhibitor cocktail (Roche Diagnostics). The protein concentration was quantified with BCA protein assay kit (Thermo Scientific, Life Technologies Ltd.). 10 *μ*g of protein was run to 4–15% mini-PROTEAN TGX precast polyacrylamide gel (Bio-Rad, Hercules, CA, USA) and transferred to PVDF membrane (Amersham Hybond-P, GE Healthcare, Little Chalfont, UK). Membranes were blocked with 5% milk for 1 h at RT and proteins were stained with MYBPC (1 : 1500, sc-166081, Santa Cruz Biotechnology), cTnT (1 : 2000, ab64623, Abcam), TPM1 (1 : 200, sc-73225, Santa Cruz Biotechnology), or *β*-actin (1 : 1000, sc-47778, Santa Cruz Biotechnology) primary antibodies over night at +4°C. Horseradish peroxidase- (HRP-) conjugated polyclonal rabbit anti-mouse (DAKO, P0260) and rabbit anti-goat IgG (Santa Cruz Biotechnology, sc-2922) were used as secondary antibodies. Stained proteins were detected by using Clarity ECL substrate (Bio-Rad) and visualized by Molecular Imager ChemiDOc XRS+ (Bio-Rad). ImageJ software (National Institutes of Health, USA) was used to compare the expression of MYBPC, cTnT, and TPM1 with the *β*-actin expression from the same cell line.

### 2.6. Statistical Analysis

For statistical analysis, control cell lines and cell lines in each mutation were combined in groups: UTA.04602.WT and UTA.04511.WT hiPSC lines in WT-CM group, UTA.02912.HCMT and UTA.13602.HCMT in HCMT-CM group, and UTA.07801.HCMM and UTA.06108.HCMM in HCMM-CM group. Mann-Whitney *U* test with Bonferroni's correction was used to analyze the differences between WT-, HCMT-, and HCMM-CMs in cell size analysis, proportion of multinucleated CMs, and Ca^2+^ imaging experiments as well as in gene expression analysis. For the statistical comparison between the three groups, one-way ANOVA followed by Tukey test was used for the patch-clamp result analysis. *p* < 0.05 was considered statistically significant. All error bars are presented as standard error of the mean (SEM).

## 3. Results

### 3.1. hiPSCs Were Derived from HCM Patients with Different Backgrounds

We derived hiPSCs from four patients carrying a HCM causing mutation either in* TPM1* (*TPM1-Asp175Asn*) or in* MYBPC3* (*MYBPC3-Gln1061X*). UTA.13602.HCMT and UTA.02912.HCMTs carry* TPM1-Asp175Asn* and UTA.07801.HCMM and UTA.06108.HCMM* MYBPC3-Gln1061X* mutation. The hiPSC lines and their mutations and abbreviations, used below, are presented in Table 2. UTA.13602.HCMT (46, XX) is derived from a 48-year-old female, whose mother died suddenly at the age of 51. Our patient has had one collapse at the age of 20 with normal heart structure, but later slight thickening of septum (16 mm) has been observed. Currently she is not on medication due to low blood pressure. UTA.02912.HCMT (46, XY) is derived from a 33-year-old male whose family member has died suddenly at the age of 21. The maximal septal thickness of our patient has been measured to be 26 mm on echocardiogram. The patient has been asymptomatic but is currently on *β*-blocker medication. UTA.07801.HCMM (46, XY) is derived from a 61-year-old male with no SCDs in the family. On echocardiogram, his myocardial septum has been observed to be 25 mm. He has atrial fibrillation and he is on *β*-blocker medication. Due to bradycardia and frequent nonsustained ventricular tachycardia episodes, an implantable cardioverter defibrillator (ICD) has been implanted. UTA.06108.HCMM (46, XY) is derived from a 55-year-old male whose father died suddenly at the age of 36 and uncle at the age of 38. Our patient has been asymptomatic with maximal septal thickness of 22 mm on echocardiogram. He is not on medication due to low blood pressure. Control hiPSC lines were derived from healthy individuals: UTA.04602.WT (46, XX) from a 56-year-old female and UTA.04511.WT (46, XY) from a 34-year-old male.

**Figure 1:**
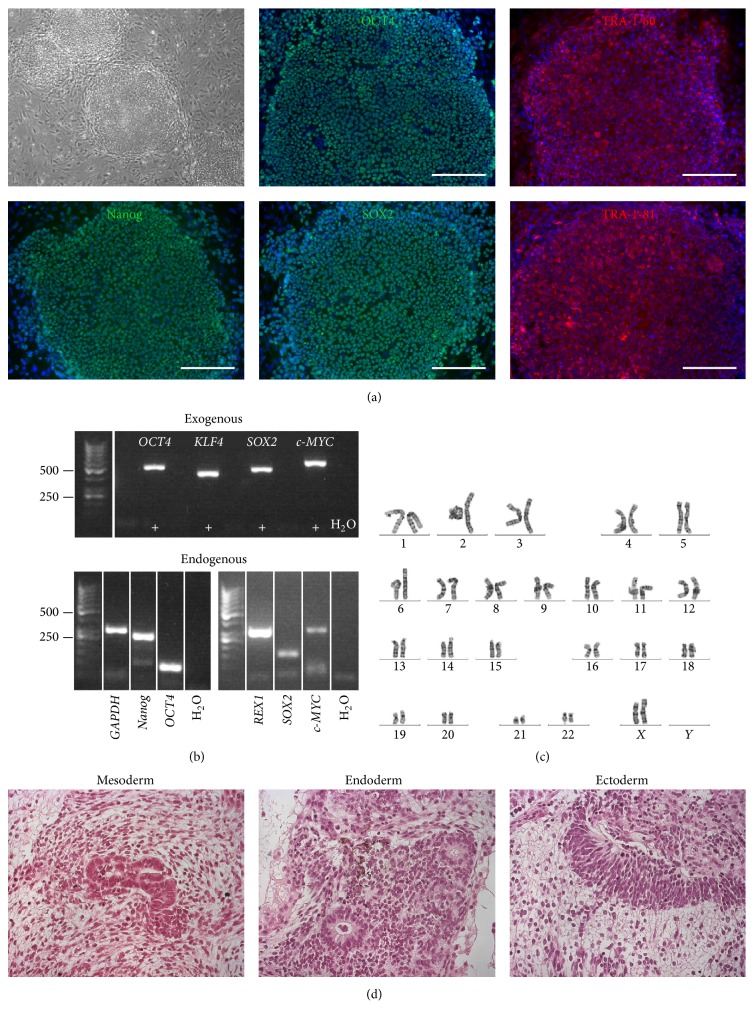
Characterization of UTA.13602.HCMT cell line. (a) The hiPSCs formed colonies expressing Nanog, OCT4, SOX2, TRA-1-60, and TRA-1-81. Scale bars: 200 *μ*m. (b) The virally transferred Sendai exogenes,* exo-OCT4* (483 bp),* exo-KLF4* (410 bp),* exo-SOX2* (451 bp), and* exo-c-MYC* (532 bp), were silenced in the hiPSCs. + indicates positive controls, for which RNA was extracted from cells 1 week after transduction. hiPSCs expressed endogenous* Nanog* (287 bp),* OCT4* (144 bp),* REX1* (306 bp),* SOX2* (151 bp), and* c-MYC* (328 bp).* GAPDH* (302 bp) was used as a housekeeping control. (c) The hiPSC line was karyotypically normal, 46 XX. (d) The pluripotency of hiPSCs was confirmed by* in vivo* teratoma assay, in which hiPSCs formed all three germ layers (mesoderm, endoderm, and ectoderm).

The pluripotent characteristics of the hiPSC-lines used were assessed (Figure 1 and Supplementary Figures 1–5). UTA.04602.WT cell line has been characterized earlier [22]. All the lines formed colonies, which expressed proteins and genes typical for hPSCs. The virally transferred exogenous genes were silenced and karyotypes of the hiPSC lines were normal. The pluripotency of hiPSC lines was proven* in vitro* by EB formation or* in vivo* by teratoma formation. The presence of* TPM1-Asp175Asn* and* MYBPC3-Gln1061X* mutations in the patient-specific hiPSC lines was confirmed by custom TaqMan SNP Genotyping Assays (Supplementary Figure 6).

### 3.2. Mutation-Specific HCM Phenotypes Were Observed in hiPSC-Derived Cardiomyocytes

**Figure 2:**
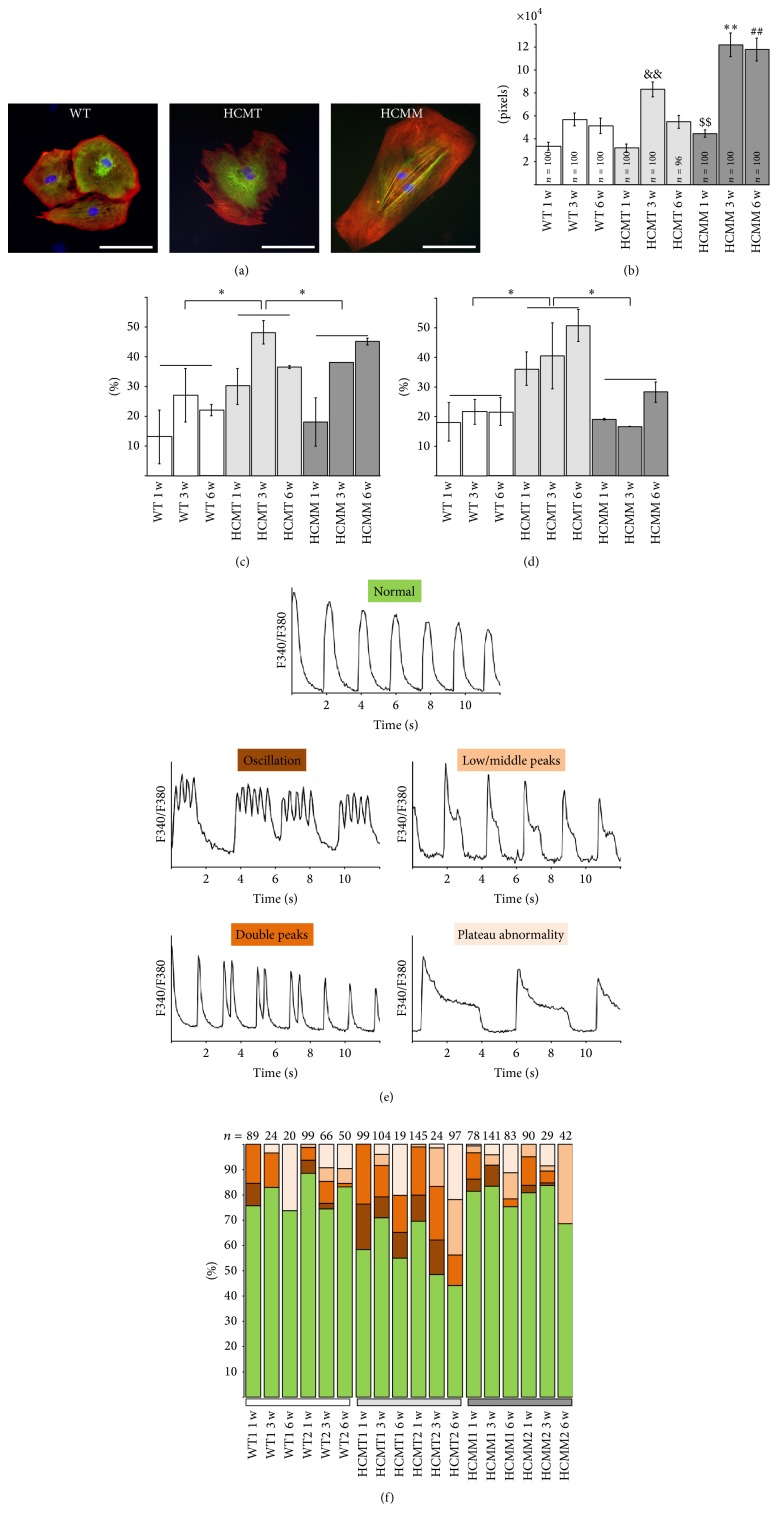
The cell size and Ca^2+^ handling of hiPSC-derived CMs after 1-, 3-, and 6-week culture as single cells. (a) Representative images of WT-CMs (WT), HCMT-CMs (HCMT), and HCMM-CMs (HCMM) stained with antibodies for cTnT (red) and MYBPC (green) proteins. Scale bars are 100 *μ*m. (b) The size of the HCMM-CMs was significantly larger in all three time points when compared to WT- and HCMT-CMs (^$$^ *p* < 0.005 when compared to WT-CMs or HCMT-CMs in the 1-week time point, ^*∗∗*^ *p* < 0.005 when compared to WT-CMs or HCMT-CMs in 3-week time point, and ^##^ *p* < 0.005 when compared to WT-CMs or HCMT-CMs in 6-week time point). HCMT-CMs were significantly larger than WT-CMs in 3-week time point (^&&^ *p* < 0.005 when compared to WT-CMs. *n* = 100, except in HCMT 6 w *n* = 96.) (c) The proportion of the multinucleated CMs was significantly higher in HCMT-CMs than in WT-CMs and HCMM-CMs when both cell lines and all time points were combined for each group (in statistical analysis *n* = 6, ^*∗*^ *p* < 0.05). The averages of multinucleated CMs were determined from the same cells, whose sizes and *n* numbers are presented in (b). (d) Significantly more CMs with Ca^2+^ handling abnormalities were observed in HCMT-CMs than in WT-CMs and HCMM-CMs when both cell lines and all time points were combined for each group (in statistical analysis *n* = 6, ^*∗*^ *p* < 0.05). The proportions of CMs with abnormalities in their Ca^2+^ handling were determined from the same Ca^2+^ imaging results presented in (f). The total *n* numbers of the analyzed CMs are presented in (f). (e) Representative images of Ca^2+^ rhythm categories. (f) Distributions of hiPSC-derived CMs in different Ca^2+^ rhythm categories (e) in each time point. WT1 = UTA.04602.WT, WT2 = UTA.04511.WT, HCMT1 = UTA.02912.HCMT, HCMT2 = UTA.13602.HCMT, HCMM1 = UTA.07801.HCMM, and HCMM2 = UTA.06108.HCMM.

All the cell lines used in the present study differentiated into cardiomyocytes similarly. Beating aggregates were formed 14–20 days after the initiation of coculturing with END-2 cells and there was no difference between the lines when the beating areas appeared. After cardiac differentiation, beating clusters were dissociated into single cells and cultured for 1, 3, and 6 weeks. The differences in the cell sizes and Ca^2+^ handling properties between two different mutations and control cells were analyzed in each time point. When comparing different types of CMs, HCMM-CMs were significantly larger than HCMT-CMs and WT-CMs in all three time points (Figures 2(a) and 2(b), *n* = 96–100, *p* < 0.005 in all cases). The enlargement of HCMT-CMs was detected after three weeks of culture when they were significantly larger than WT-CMs (*p* < 0.005). Generally, the size of the hiPSC-derived CMs in all groups increased when the cells cultured for three weeks. Within each group, there were no differences in cell sizes between three and six weeks, except with HCMT-CMs, which seemed to be smaller in size 6-week time point. However, during 6 weeks of culture, cell types other than CMs had the tendency to overgrow the CM culture, which might have affected the cellular enlargement. This phenomenon was observed especially with UTA.13602.HCMT cell line (data not shown). When all time points in each group (WT-CM, HCMT-CM, and HCMM-CM) were combined, the number of multinucleated CMs was significantly higher in HCMT-CMs than in WT-CMs and HCMM-CMs (*n* = 6 in statistical analysis, *p* < 0.05, Figure 2(c)).

The Ca^2+^handling properties of hiPSC-derived CMs were studied by Ca^2+^ imaging. HCMT-CMs had significantly more abnormalities than WT-CMs and HCMM-CMs, when all time points in each group were combined (Figure 2(d), *n* = 6 in statistical analysis, *p* < 0.05). Spontaneously beating CMs were divided into five different rhythm categories (normal, oscillation, low/middle peaks, double peaks, and plateau abnormality) based on the abnormalities observed in their Ca^2+^ signals (Figures 2(e) and 2(f)). HCMT-CMs had significantly higher number of double peaks than HCMM-CMs (*p* < 0.05, *n* = 6 in statistical analysis) when both cell lines and all time points were combined for each group.

### 3.3. Action Potential Characteristics of WT and HCM hiPSC-Derived Cardiomyocytes

**Figure 3:**
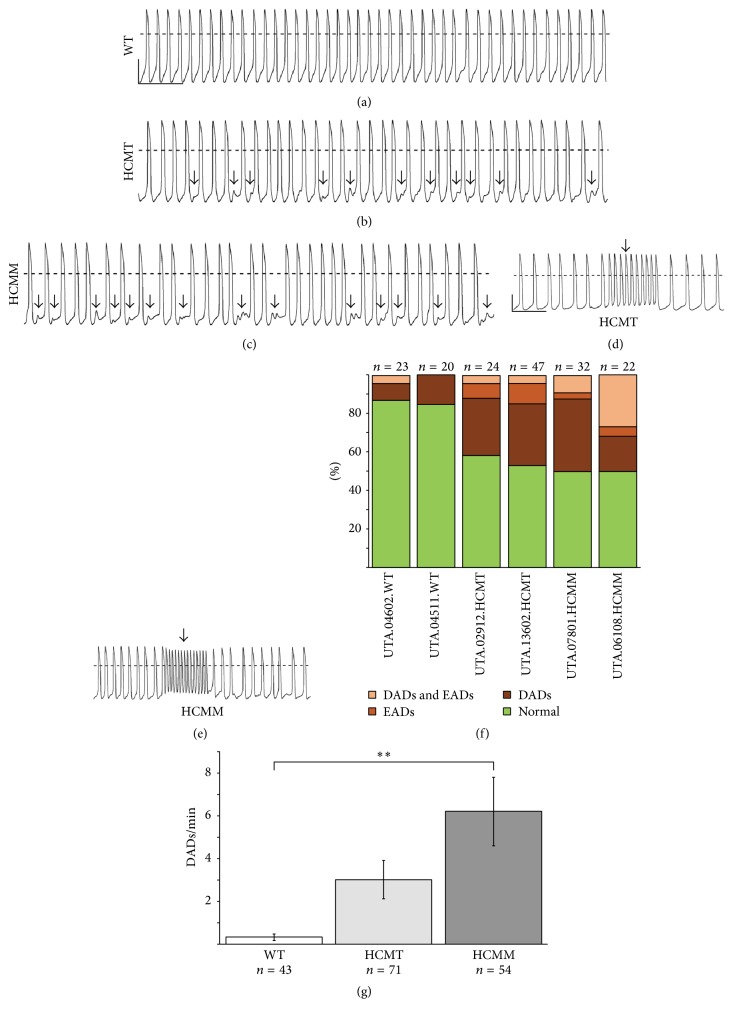
Arrhythmogenic events (DADs and EADs) were observed in HCM-CMs. (a)–(e) Representative recordings of control hiPSC-derived CMs (WT) and hiPSC-derived CMs carrying* TPM1-Asp175Asn* (HCMT) or* MYBPC3-Gln1061X* (HCMM) mutations. Typical DADs (arrows) are presented in (b) and (c) and EADs (arrows) in (d) and (e) for HCMT-CMs and HCMM-CMs, respectively. Scale bars represent 40 mv and 5 seconds, respectively. Scale bars in (a) are representative for (b) and (c), and scale bars in (d) are representative for (e). (f) Distribution of CMs exhibiting arrhythmogenic events in each cell line. (g) DAD rate was significantly higher in HCMM-CMs than in WT-CMs (^*∗∗*^ *p* < 0.005).

The spontaneous action potentials were recorded from the beating hiPSC-derived CMs to establish the electrophysiological baselines. Most of the cells (>80%) were ventricular-like CMs in all the hiPSC lines studied. For this reason, only ventricular-like waveforms are presented here. We first analyzed the percentage of the arrhythmias in each cell line (Figures 3(a)–3(f)) and found similar percentage in both cell lines within the groups (UTA.04602.WT (13%) versus UTA.04511.WT (15%), UTA.02912.HCMT (42%) versus UTA.13602.HCMT (47%), and UTA.07801.HCMM (50%) versus UTA.06108.HCMM (50%)). Based on the percentage of the arrhythmias, we combined hiPSC-derived CMs into groups (WT-CM, HCMT-CM, and HCMM-CM) for further analysis.

Both HCMT-CMs and HCMM-CMs had more arrhythmic events including delayed after depolarizations (DADs) and early after depolarizations (EADs) when compared to the WT-CMs (WT-CM (14%), HCMT-CM (45%), and HCMM-CM (50%)). We quantified the occurrence of DADs in hiPSC-derived CMs as a rate (DADs/min) calculated as total number of DADs/total number of APs. We found that the DAD rate in HCMM-CMs was significantly higher than in WT-CMs (Figure 3(g), *p* < 0.005).

The average APD at 50% repolarization (APD_50_) and 90% repolarization (APD_90_) of HCMT-CMs was significantly longer than those of the WT-CMs (APD_50_ (*p* < 0.005) and APD_90_ (*p* < 0.005)) and HCMM-CMs (APD_50_ (*p* < 0.05) and APD_90_ (*p* < 0.05)) (Table 3). APD_90_ of HCMM-CMs was significantly longer than that of the WT-CMs (*p* < 0.05) (Table 3). Furthermore, the beating rates of both HCMT-CMs and HCMM-CMs were significantly lower than in WT-CMs (WT-CM versus HCMT-CM (*p* < 0.005) and WT-CM versus HCMM-CM (*p* < 0.005)). In addition, the APA of HCMM-CMs was significantly higher than in the WT-CMs (*p* < 0.05). However, no significant differences were found for the MDP between any groups (Table 3).

### 3.4. Differences in the Gene Expression Profiles of hiPSC-Derived Cardiomyocytes

**Figure 4:**
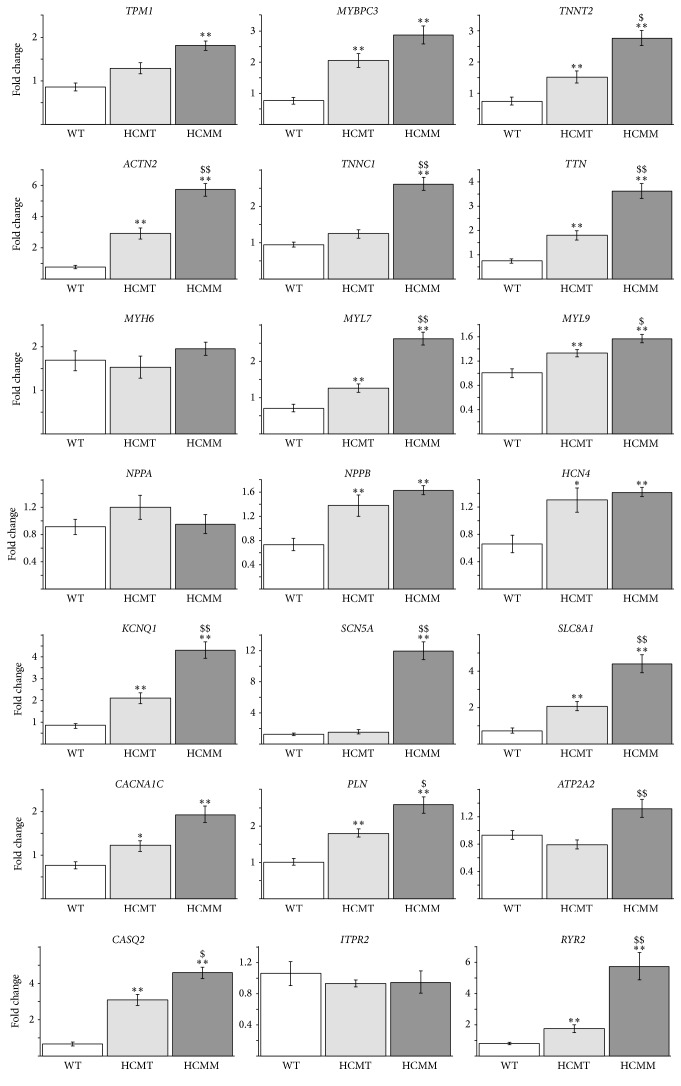
Gene expression profiles in control hiPSC-derived CMs (WT) and in hiPSC-derived CMs carrying* TPM1-Asp175Asn* (HCMT) or* MYBPC3-Gln1061X* (HCMM) mutations. (^*∗*^ represents HCMT or HCMM versus WT, and ^$^ represents HCMT versus HCMM. ^*∗∗*^ or ^$$^ *p* < 0.005, ^*∗*^ or ^$^ *p* < 0.05  *n* = 16, except for* NPPA n* = 8).

Dissociated hiPSC-derived CMs were cultured for one week before qRT-PCR analysis was performed. The results are presented in Figure 4. The expression of sarcomeric genes* MYBPC3*,* TNNT2*, * ACTN2*,* TTN*,* MYL7*, and* MYL9* was significantly higher in both HCMT-CMs and HCMM-CMs than in the WT-CMs (*p* < 0.005 in all cases). The expression of* TPM1* and* TNNC1* was significantly increased only in the HCMM-CMs when compared to WT-CMs (*p* < 0.005 in both cases). On the other hand, the expression of* MYH6* was on the same level in all hiPSC-derived CMs. Moreover, the expression of some sarcomeric genes (*TNNT2*, * ACTN2*,* TNNC1*,* TTN*,* MYL7*, and* MYL9*) was significantly higher in the HCMM-CMs than in the HCMT-CMs (*p* < 0.005 in other than* TNNT2* and* MYL9 p* < 0.05). The expression of natriuretic peptide A (*NPPA*) was similar in all hiPSC-derived CMs while the expression of natriuretic peptide B (*NPPB*) was increased in HCMT-CMs and HCMM-CMs when compared to WT-CMs (*p* < 0.005 in both cases). Nodal marker* HCN4* was also significantly increased in both HCM-CMs when compared to WT-CMs (*p* < 0.05 for HCMT-CMs versus WT-CMs and *p* < 0.005 for HCMM-CMs versus WT-CMs). Further, the highest expression of potassium channel* KCNQ1* and sodium channel* SCN5A* as well as sodium calcium exchanger* SLC8A1* was observed in HCMM-CMs (*p* < 0.005 when compared to WT-CMs in all cases).

We found differences also in the expression of genes related to the Ca^2+^ handling. The expression of* CACNA1C* and* PLN* was increased in both HCMT-CMs (*p* < 0.05 for* CACNA1C* and *p* < 0.005 for* PLN*) and HCMM-CMs (*p* < 0.005 in both cases) when compared to WT-CMs while the expression of* ATP2A2* and* ITPR2* was on the same level in all hiPSC-derived CMs. However, the expression of* CASQ2* and* RYR2* was significantly higher in HCMT-CMs and HCMM-CMs when compared to WT-CMs (*p* < 0.005 in all cases). Moreover, the expression of* RYR2* was almost six times higher in the HCMM-CMs than in the WT-CMs (*p* < 0.005) and around three times higher than in HCMT-CMs (*p* < 0.005).

### 3.5. Truncated MYBPC Protein Was Not Detected in hiPSC-Derived Cardiomyocytes Carrying MYBPC3-Gln1061X Mutation

**Figure 5:**
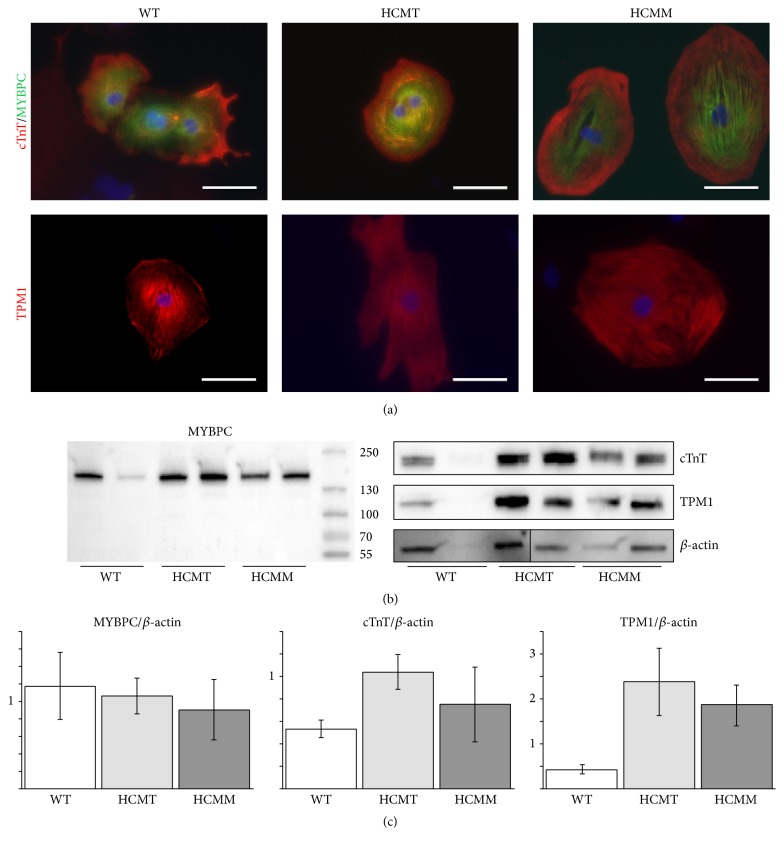
Cardiac-specific protein expression in hiPSC-derived CMs. (a) Representative images of hiPSC-derived CMs carrying* TPM1-Asp175Asn* mutation (HCMT) or* MYBPC3-Gln1061X* mutation (HCMM) and hiPSC-derived control CMs (WT) stained with cTnT, MYBPC, and TPM1. These images are not to quantify the protein expression but to demonstrate the presence of cTnT, MYBPC, and TPM1 proteins in the hiPSC-derived CMs. (b) hiPSC-derived CMs from all cell lines expressed MYBPC, cTnT, and TPM1 proteins. The truncated MYBPC was not detected in HCMM cells (size of the wild type protein 141 kDa and the predicted size of the truncated protein 117 kDa). (c) The expression of MYBPC, cTnT, and TPM1 in WT-CMs, HCMT-CMs, and HCMM-CMs normalized to the expression of *β*-actin. Protein expressions were quantified from western blots using ImageJ software. Quantitation data show the averages of MYBPC/*β*-actin, cTnT/*β*-actin, and TPM1/*β*-actin relations from hiPSC-derived CMs from two different hiPSC lines in each group. Because of the lack of replicates, statistical analysis was not performed.

Both wild type and the mutant* TPM1* mRNA were present in HCM-CMs carrying the* TPM1-Asp175Asn* mutation while hiPSC-derived control CMs expressed only wild type mRNA. However, in hiPSC-CMs carrying the* MYBPC3-Gln1061X* mutation the mutant mRNA was not clearly detected (Supplementary Figure 7). At a protein level all hiPSC-derived CMs expressed MYBPC, cTnT, and TPM1 (Figures 5(a) and 5(b)). The truncated MYBPC protein (predicted size: 116 kDa) was not detected in HCMM-CMs with western blot analysis (Figure 5(b)). However, the expression level of total MYBPC was slightly reduced in HCMM-CMs when compared to WT-CMs (Figure 5(c)). In addition, the expression of cTnT and TPM1 was elevated in both HCM-CMs (Figure 5(c)).

## 4. Discussion

Here we have analyzed characteristics of hiPSC-derived HCM-CMs carrying either* TPM1-Asp175Asn* or* MYBPC3-Gln1061X* gene mutation. The size of the* MYBPC3-Gln1061X* CMs was significantly larger than that of* TPM1-Asp175Asn* CMs, while the CMs carrying the latter mutation had significantly more abnormal Ca^2+^ transients. Additionally, CMs with* TPM1-Asp175Asn* mutation had significantly more prolonged action potentials. However, both types of HCM-CMs had increased amount of arrhythmogenic events (DADs and EADs) in electrophysiological recordings when compared to control CMs. In addition to morphological and functional differences, also gene expression profiles were different between CMs carrying either* TPM1-Asp175Asn* or* MYBPC3-Gln1061X* gene mutation.

Genetic HCM is primarily due to mutations in sarcomeric genes, while changes at the cellular level include disturbed Ca^2+^ metabolism and decreased contraction force generation in addition to enlarged cell size [23]. To our knowledge, total of three reports studying the characteristics as well as the pathophysiological mechanisms of the HCM by using the patient-specific hiPSCs have been published [12–14]. In two of these publications, the mutation is located in the* MYH7* (*MYH7-R663H* or* MYH7-R442G*) [12, 13], whereas, in the most recent publication, the hiPSCs were derived from three HCM patients, from whom one carried the* MYBPC3-999-1004del2*, while the other mutations were unknown [14]. In addition to these three publications, hiPSC-derived CMs carrying* MYBPC3* mutations have been used in one study where the effects of serum on the phenotype of neonatal rat CMs as well as hPSC-derived CMs have been explored [24]. hiPSC-derived CMs carrying* MYBPC3* mutation were used only when studying the effects of serum on the cellular enlargement [24]. In this current study, we obtained hiPSCs from HCM patients carrying either the* MYBPC3-Gln1061X* or* TPM1-Asp175Asn* mutation. We believe that this is the first report where hiPSC-derived CMs carrying different gene mutations have been compared in the same study with similar experimental settings.

Our HCM-CMs demonstrated cellular enlargement similarly to previous HCM studies with hiPSCs [12–14]. However, we observed a significant difference in the cellular enlargement between the two HCM mutations. CMs carrying the* MYBPC3-Gln1061X* mutation presented more pronounced and earlier cellular enlargement than CMs carrying the* TPM1-Asp175Asn* mutation. In a previous study, serum has been shown to mask hypertrophic phenotype of the CMs with mutations in the* MYBPC3* [24]. The CMs from the HCM patients were larger in serum-free conditions without any external stimuli, while the serum seemed to increase the cellular enlargement in WT-CMs but not in CMs with HCM mutations [24]. We used 20% serum in our CM culture medium that did not seem to mask the cellular enlargement with our CMs. Already after one week of culture, CMs carrying* MYBPC3-Gln1061X *mutation were significantly larger than the WT-CMs. The enlargement of CMs carrying* TPM1-Asp175Asn* mutation was detectable only after three weeks of culture. In the previous clinical studies, as well as in the patient data analyzed in this current study, the hypertrophy has been in the same range in patients carrying either of these two mutations [2]. Therefore, these differences in CM size between the two mutations do not correlate with the extent of clinical hypertrophy.

The higher Ca^2+^ sensitivity, observed in animal models and myectomy samples, has been suggested to be a common feature for all HCM mutations [25, 26]. In the previous studies with hiPSC-derived CMs, irregularities in Ca^2+^ transients have been observed in the* MYH7-R663H* and the* MYH7-R442G* mutations [12, 13]. The higher Ca^2+^ sensitivity has been related to lower phosphorylation levels of the MYBPC and the Troponin I proteins and the difference could at least partly be explained by hypophosphorylation of the sarcomeric proteins compared to the actual mutations [26]. In our study, the amount of abnormalities in Ca^2+^transients was significantly increased only in the hiPSC-derived CMs with the* TPM1-Asp175Asn* mutation. Indeed, the amount of irregularities in Ca^2+^ handling properties was similar in the* MYBPC3-Gln1061X* compared to that in the WT-CMs. The phosphorylation of the proteins was not analyzed in this current study. However, we analyzed the expression of genes related to Ca^2+^ handling and they were at the highest level in hiPSC-derived CMs carrying the* MYBPC3-Gln1061X* mutation. This might be at least partly due to the larger cell size of these CMs. Han and coworkers found decreased level of* RYR2* expression in HCM-CMs carrying* MYH7-R442G* mutation [13], while in our study the expression of* RYR2* was significantly higher in both mutations and almost six times higher in the hiPSC-derived CMs carrying the* MYBPC3-Gln1061X* mutation than in the WT-CMs. These observations suggest that abnormal Ca^2+^ transients in HCM-CMs carrying different mutations may be caused by distinct mechanisms.

One of the fundamental features of the HCM is its association with ventricular arrhythmias responsible for severe cardiac malfunctions including sudden cardiac death [27, 28]. We found increased amount of arrhythmogenic events (DADs and EADs) in both HCM-CMs. Furthermore, decreased beating rate was observed in both types of HCM-CMs, which could be due to higher occurrence of DADs between the two consecutive APs. In addition, the APD_90_ of hiPSC-derived CMs carrying either* TPM1-Asp175Asn* or* MYBPC3-Gln1061X* mutation was longer than in WT-CMs, which is in line with previous findings with different mutations [12, 13, 29]. The mechanism of arrhythmias in HCM is not yet fully understood; however, imbalances in Ca^2+^ homeostasis are considered as a main cause of arrhythmias shown in the previous studies [12, 13]. Clinically, patients carrying* TPM1-Asp175Asn* mutation have been reported to be more prone to arrhythmias than those carrying* MYBPC3-Gln1061X* mutation [30]. Our data with hiPSC-derived CMs support this finding by demonstrating more abnormal Ca^2+^ transients and longer APD_90_ in* TPM1-Asp175Asn* CMs than in HCM-CMs carrying the* MYBPC3-Gln1061X* mutation.

Like most of the HCM mutations located in the* MYBPC3*, also* MYBPC3-Gln1061X* is a nonsense mutation that leads to premature stop-codon [31]. Nonsense mutations are suggested to act through haploinsufficiency in which the mutated protein is either degraded or not produced at all. The truncated form of MYBPC has not been found in human cardiac samples while the total expression level of MYBPC has been reported to vary from being decreased to even increased [32–36]. Interestingly, when studying myectomy samples from HCM hearts with* MYBPC3* mutation, Helms et al. observed that the total amount of* MYBPC3* mRNA was increased, while the total amount of MYBPC protein was on the same level compared to that in the control hearts. They hypothesized that the upregulation of the* MYBPC3 *compensates the degraded truncated MYBPC protein [35]. We could not detect mutant allele on mRNA expression level or truncated MYBPC protein in hiPSC-derived CMs carrying the* MYBPC3-Gln1061X* mutation. Similar absence of truncated protein has been reported earlier with a different MYBPC mutation in hiPSC-derived CMs [14]. These data suggest that the mutant mRNA might be degraded. However, further research is still needed to confirm the results and to discover the actual degradation mechanism.

The two HCM mutations analyzed in this study are the most frequent mutations in Finland accounting for about 18% of all Finnish HCM patients [3]. In our study, we found differences in the morphological and biochemical properties, as well as in Ca^2+^ cycling and electrophysiological properties between the CMs carrying either* TPM1-Asp175Asn* or* MYBPC3-Gln1061X* mutation. However, we have not analyzed the possible effects of other gene mutations and epigenetic factors on the phenotype differences observed in our study. Additionally, we used only two hiPSC lines from two different patients in each mutation in our experiments. In the future, we need to extend our studies including further patients and studying the effects of additional gene mutations and epigenetic factors. The immature nature of hiPSC-derived CMs is a general limitation when using these cells in disease modeling. Further development in the differentiation and maturation protocols will increase the reliability of studies conducted with hiPSC-derived CMs. Finally, the exact pathophysiology in abnormal Ca^2+^ transients or electrical abnormalities is not known and further research with combined patch clamp and Ca^2+^ imaging is required in the future to reveal the significance of the cellular findings for clinical phenotypes as well as for treatment options.

## 5. Conclusions

In conclusion, both HCM hiPSC-derived CMs either carrying the* TPM1-Asp175Asn* or* MYBPC3-Gln1061X* mutation exhibited pathological changes related to HCM. However, significant differences between the two mutations were observed. The hiPSC-derived cell models, established in this study, can be exploited to study further the pathophysiological mechanisms of HCM as well as to screen drugs and potentially optimize treatments in mutation-specific way.

## Supplementary Material

Supplementary Figure 1: Characterization of UTA.04511.WT cell line.
Supplementary Figure 2: Characterization of UTA.02912.HCMT cell line.
Supplementary Figure 3: Characterization of UTA.07801.HCMM cell line.
Supplementary Figure 4: Characterization of UTA.06108.HCMM cell line.
Supplementary Figure 5: Karyotype analyses of the cell lines A. UTA.04511.WT and B. UTA.02912.HCMT.
Supplementary Figure 6: The presence of the TPM1-Asp175Asn and MYBPC3-Gln1061X mutation in the patient-specific hiPSC lines was confirmed by custom TaqMan SNP Genotyping Assays.
Supplementary Figure 7: The mRNA expression of mutant and wildtype allelesin the hiPSC-derived CMs carrying TPM1-Asp175Asn or MYBPC3-Gln1061X mutation were assessed with the TaqMan SNP Genotyping Assays.
Supplementary Table 1: Sequences of the primers and probes (Custom TaqMan SNP Genotyping Assays, Applied Biosystems, Life Technologies Ltd) used in the genotyping and mutant allele mRNA expression assays.

## Figures and Tables

**Table 1:** TaqMan assays used in qRT-PCR experiments.

Gene	Description/alias	Function	TaqMan assay ID
*EEF1A1*	Eukaryotic translation elongation factor 1 alpha 1	Housekeeping gene	Hs00265885_g1
*GAPDH*	Glyceraldehyde-3-phosphate dehydrogenase	Housekeeping gene	Hs02758991_g1
*TNNT2*	Troponin T	Sarcomeric gene	Hs00165960_m1
*MYH6*	Myosin heavy chain 6	Sarcomeric gene	Hs01101425_m1
*ACTN2*	*α*-actinin 2	Sarcomeric gene	Hs00153809_m1
*TPM1 *	*α*-tropomyosin	Sarcomeric gene	Hs00165966_m1
*MYBPC3*	Myosin-binding protein C	Sarcomeric gene	Hs00165232_m1
*TTN*	Titin	Sarcomeric gene	Hs00399225_m1
*TNNC1*	Troponin C type 1	Sarcomeric gene	Hs00896999_g1
*MYL9*	Myosin, light chain 2/MLC2	Sarcomeric gene	Hs00697086_m1
*MYL7*	Myosin, light chain 7	Sarcomeric gene	Hs01085598_g1
*NPPA*	Natriuretic peptide A	Hypertrophy marker	Hs01081097_m1
*NPPB*	Natriuretic peptide B	Hypertrophy marker	Hs01057466_g1
*HCN4*	Hyperpolarization activated cyclic nucleotide-gated potassium channel 4	Potassium channel	Hs00975492_m1
*KCNQ1*	Voltage-gated potassium channel, KQT-like subfamily, member 1	Potassium channel	Hs00923522_m1
*CACNA1C*	Voltage-dependent calcium channel, L type, alpha 1C subunit/CaCNA1.2	Calcium channel	Hs00167681_m1
*SCN5A*	Voltage-gated sodium channel, V type, alpha subunit	Sodium channel	Hs00165693_m1
*SLC8A1*	Solute carrier family 8, member 1/NCX1	Na^+^/Ca^2+^ exchanger	Hs01062258_m1
*PLN*	Phospholamban/PLB	Protein kinase substrate	Hs01848144_s1
*ATP2A2*	ATPase, Ca^2+^ transporting, cardiac muscle, slow twitch 2/SERCA2a	Ca^2+^-ATPase	Hs00544877_m1
*CASQ2*	Calsequestrin	Ca^2+^ binding protein in SR	Hs00154286_m1
*ITPR2*	Inositol 1,4,5-trisphosphate receptor, type 2/IP3R2	Ca^2+^ receptor	Hs00181916_m1
*RYR2*	Ryanodine receptor 2 (cardiac)	Ryanodine receptor	Hs00892883_m1

**Table 2:** The hiPSC lines and their mutations and abbreviations used in the study.

Group	Cell line	Mutation	Name in Figure 2(f)
WT	UTA.04602.WT	—	WT1
	UTA.04511.WT	—	WT2

HCMT	UTA.02912.HCMT	*TPM1-Asp175Asn*	HCMT1
	UTA.13602.HCMT	*TPM1-Asp175Asn*	HCMT2

HCMM	UTA.07801.HCMM	*MYBPC3-Gln1061X*	HCMM1
	UTA.06108.HCMM	*MYBPC3-Gln1061X*	HCMM2

**Table 3:** AP properties of ventricular-like CMs derived from control hiPSC lines (WT) and from hiPSC lines carrying *TPM1-Asp175Asn* (HCMT) or *MYBPC3-Gln1061X* (HCMM) mutations. In the results, the data of each group is comprised from two separate cell lines.

Group	*n*	Beating rate	APD_50_	APD_90_	APA	MDP
		(BPM)	(ms)	(ms)	(mV)	(mV)
WT	43	58.1 ± 2.3	277.3 ± 13.0	323.6 ± 13.9	119.5 ± 1.1	−76.8 ± 0.8
HCMT	71	48.4 ± 1.5^*∗∗*^	372.3 ± 13.2^*∗∗*^	433.1 ± 14.0^*∗∗*^	121.2 ± 1.1	−75.8 ± 0.7
HCMM	54	47.1 ± 1.8^*∗∗*^	319.5 ± 13.7^$^	377.6 ± 15.0^*∗*,$^	124.3 ± 1.4^*∗*^	−77.9 ± 0.8

^*∗*^HCMT or HCMM versus WT.

^$^HCMM versus HCMT.

^$^ or ^*∗*^ *p* < 0.05 and ^*∗∗*^ *p* < 0.005.

## Abbreviations

AP: Action potential
APA: Action potential amplitude
APD: Action potential duration
bFGF: Basic fibroblast growth factor
BPM: Beats per minute
CM: Cardiomyocyte
CPVT: Catecholaminergic polymorphic ventricular tachycardia
cTnT: Troponin T
DAD: Delayed after depolarization
DAPI: 40,6-Diamidino-2-phenylindole
DCM: Dilated cardiomyopathy
EAD: Early after depolarization
EB: Embryoid body
END-2: Mouse endodermal-like cells
FBS: Fetal bovine serum
HCM: Hypertrophic cardiomyopathy
HCM-CM: Cardiomyocytes derived from hiPSCs carrying HCM mutation
HCMM-CM: Cardiomyocytes derived from hiPSCs carrying* MYBPC3-Gln1061X* mutation
HCMT-CM: Cardiomyocytes derived from hiPSCs carrying* TPM1-Asp175Asn* mutation
hiPSC: Human induced pluripotent stem cell
hPSC: Human pluripotent stem cell
HRP: Horseradish peroxidase
ICD: Implantable cardioverter defibrillator
ko: Knockout
ko-SR: Knockout serum replacement
MDP: Maximum diastolic potential
MEF: Mouse embryonic fibroblast
MYBPC: Myosin-binding protein C
MYH7: *β*-myosin heavy chain
NEAA: Nonessential amino acids
RT-STA: Reverse transcription-specific target amplification
SCD: Sudden cardiac death
SEM: Standard error of the mean
TPM1: *α*-tropomyosin
WT-CM: Cardiomyocytes derived from control hiPSCs.
