# Supplementary material for: Mutation-Specific Phenotypes in hiPSC-Derived Cardiomyocytes Carrying Either Myosin-Binding Protein C Or α-Tropomyosin Mutation for Hypertrophic Cardiomyopathy
Source: Stem Cells Int. 2015 Dec 28;2016:1684792. doi: 10.1155/2016/1684792 (PMC4707351; doi:10.1155/2016/1684792)
Supplement: Supplementary file 1 — Supplementary Figure 1: Characterization of UTA.04511.WT cell line. Supplementary Figure 2: Characterization of UTA.02912.HCMT cell line. Supplementary Figure 3: Characterization of UTA.07801.HCMM cell line. Supplementary Figure 4: Characterization of UTA.06108.HCMM cell line. Supplementary Figure 5: Karyotype analyses of the cell lines A. UTA.04511.WT and B. UTA.02912.HCMT. Supplementary Figure 6: The presence of the TPM1-Asp175Asn and MYBPC3-Gln1061X mutation in the patient-specific hiPSC lines was confirmed by custom TaqMan SNP Genotyping Assays. Supplementary Figure 7: The mRNA expression of mutant and wildtype allelesin the hiPSC-derived CMs carrying TPM1-Asp175Asn or MYBPC3-Gln1061X mutation were assessed with the TaqMan SNP Genotyping Assays. Supplementary Table 1: Sequences of the primers and probes (Custom TaqMan SNP Genotyping Assays, Applied Biosystems, Life Technologies Ltd) used in the genotyping and mutant allele mRNA expression assays. [file 1684792.f1.zip › 1684792.f1/mat.746594.v3.pdf]

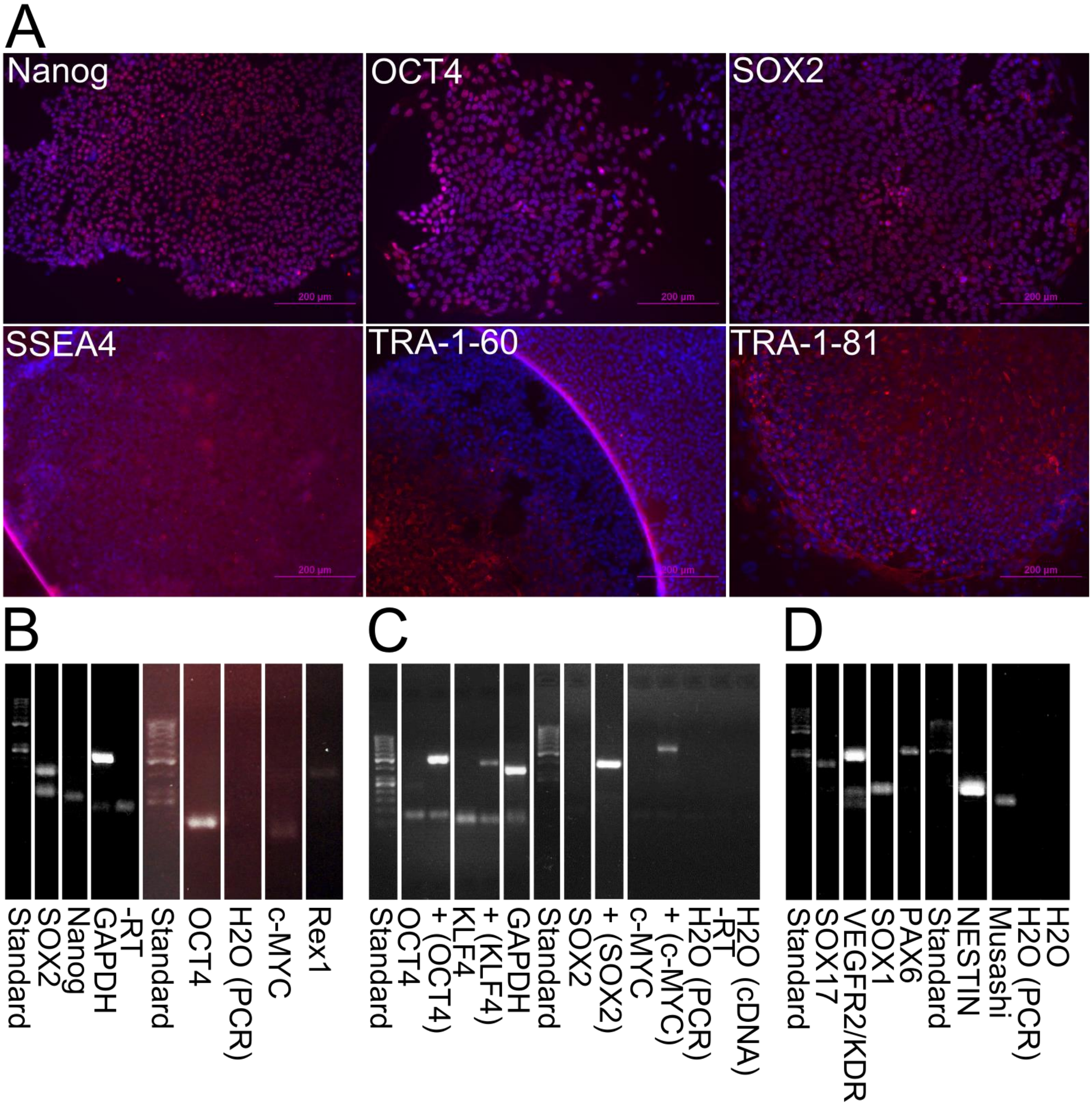

Supplementary Figure 1.

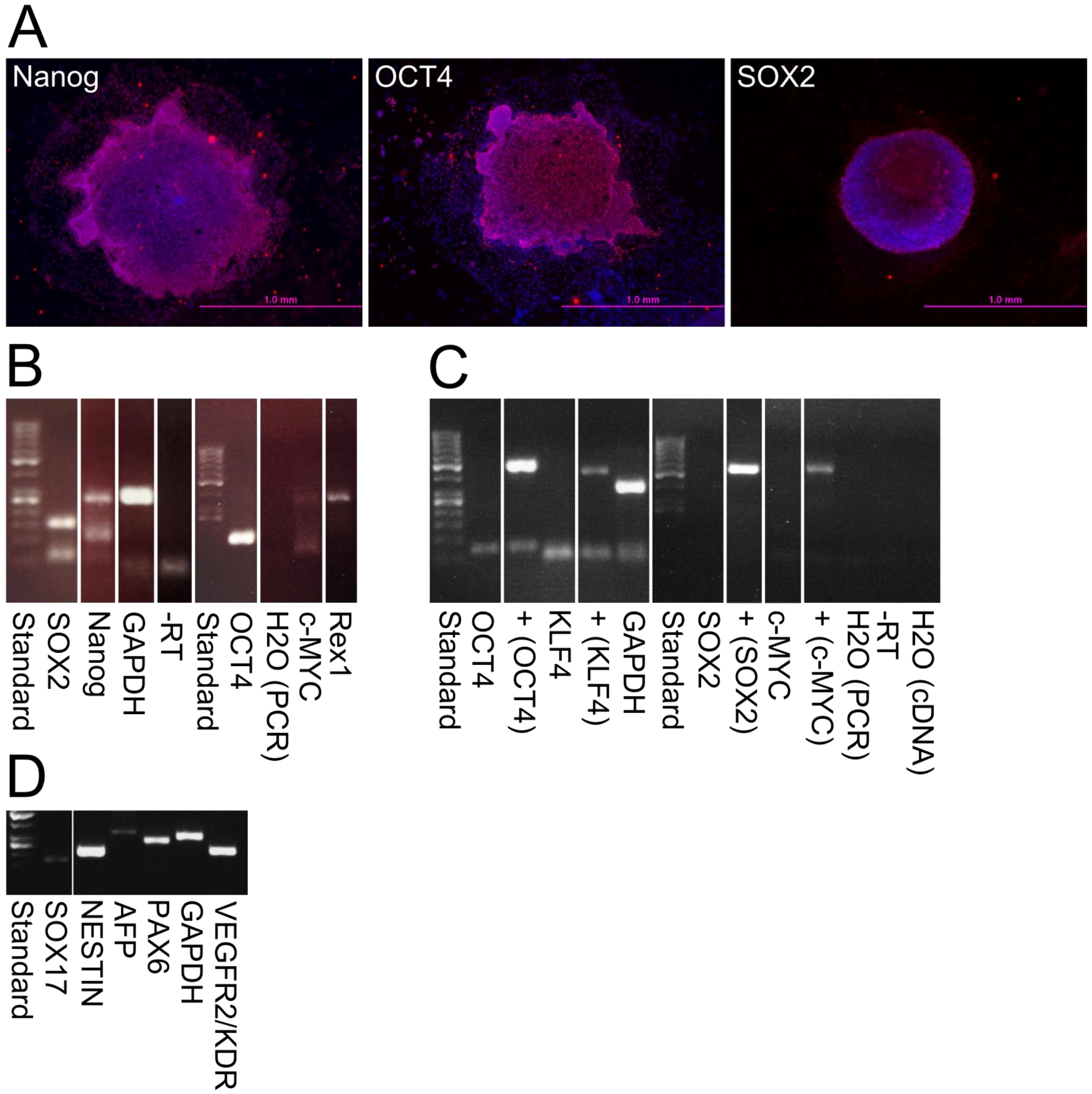

Supplementary Figure 2.

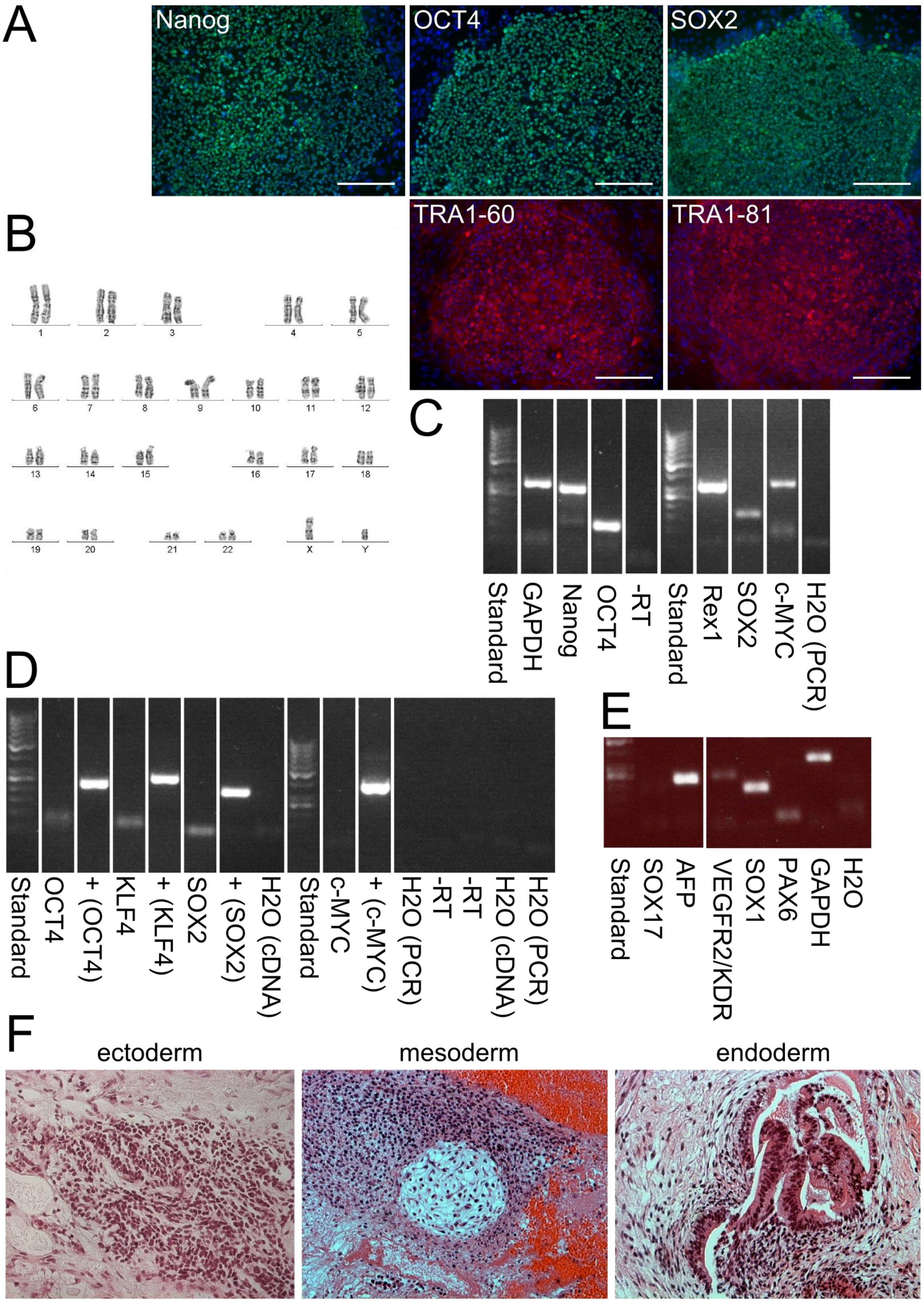

Supplementary Figure 3.

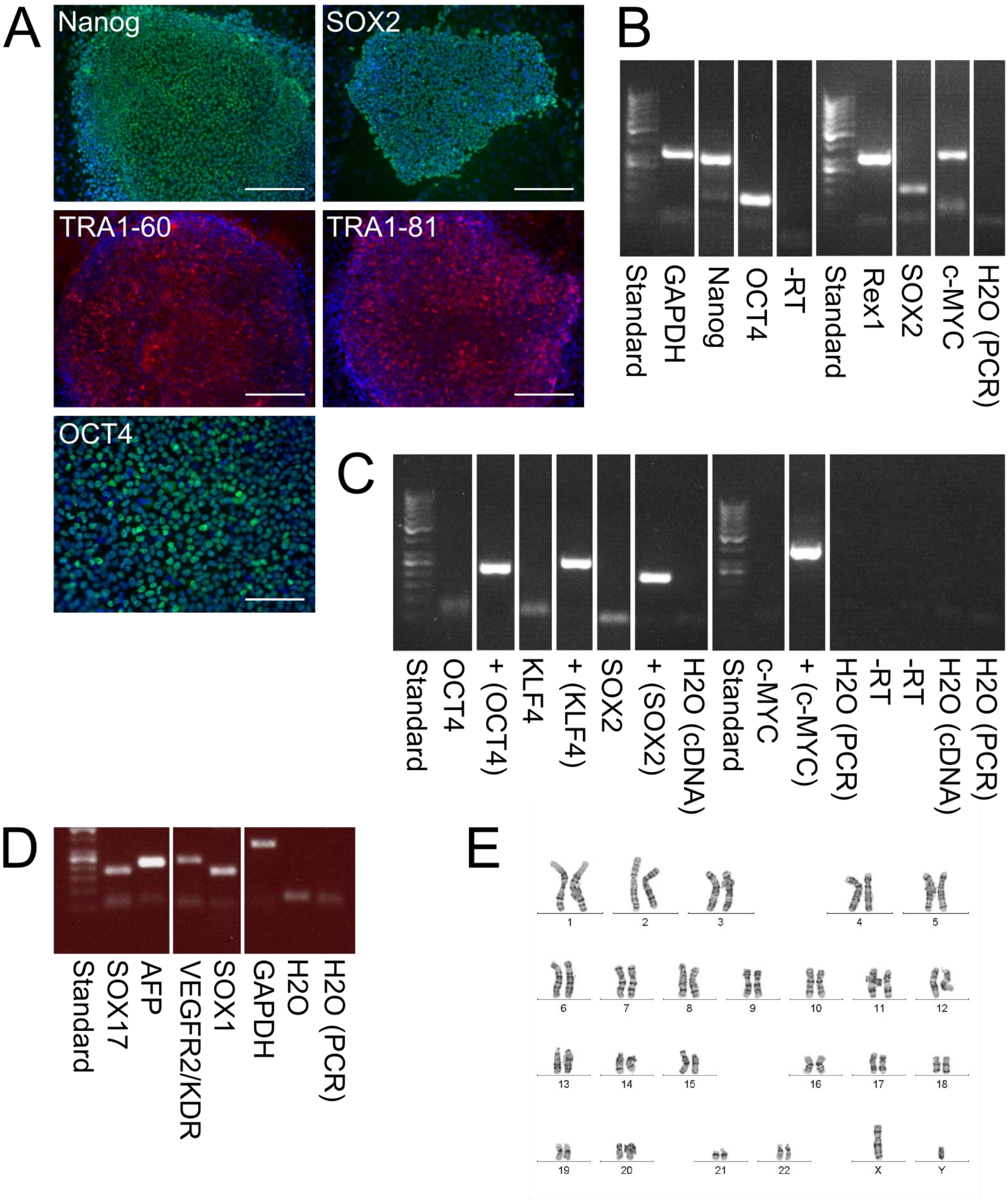

Supplementary Figure 4.

A

Normalized Ratios

Probe	/F	/M	Chromosomal Location
01C	0.98	1.00	
02C	1.00	0.99	
03C	0.98	1.02	
04C	1.00	1.03	
05C	1.07	1.04	
06C	1.02	1.04	
07C	1.06	1.04	
08C	0.97	0.96	
09C	0.97	1.01	
10C	1.02	1.00	
11C	0.93	0.97	
12C	1.02	1.01	
13C	1.02	0.99	
14C	0.96	0.99	
15C	0.99	0.99	
16C	1.02	1.03	
17C	0.98	0.98	
18C	0.98	0.98	
19C	1.01	1.03	
20C	1.03	0.97	
21C	1.03	0.99	
22C	1.00	1.05	
Auto	1.03	0.99	
X1;ptel	0.70	0.99	-
X2;pcen	0.64	0.97	-
X3;qcen	0.77	0.97	-
X4;qtel	0.71	0.93	-
Y1;ptel	1.48	0.96	-
Y2;pcen	2.50	0.99	-
Y3;qcen	1.35	1.05	-
Y4;qtel	2.18	0.99	-

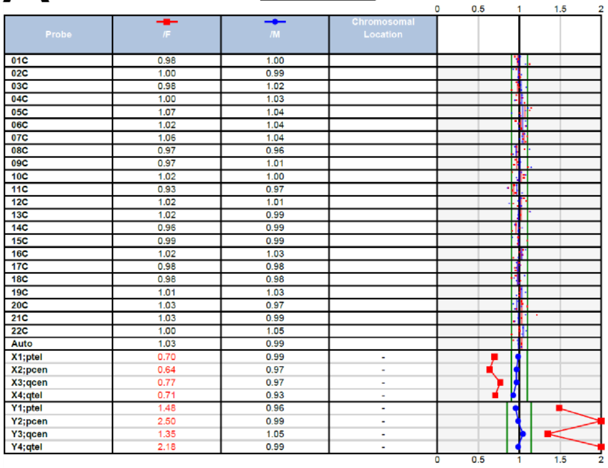

Normalized Ratios

Probe	/F	/M	Chromosomal Location
01C	0.98	1.00	
02C	1.00	0.99	
03C	0.98	1.02	
04C	1.00	1.03	
05C	1.07	1.04	
06C	1.02	1.04	
07C	1.06	1.04	
08C	0.97	0.96	
09C	0.97	1.01	
10C	1.02	1.00	
11C	0.93	0.97	
12C	1.02	1.01	
13C	1.02	0.99	
14C	0.96	0.99	
15C	0.99	0.99	
16C	1.02	1.03	
17C	0.98	0.98	
18C	0.98	0.98	
19C	1.01	1.03	
20C	1.03	0.97	
21C	1.03	0.99	
22C	1.00	1.05	
Auto	1.03	0.99	
X	0.70	0.97	
Y	1.83	0.99	

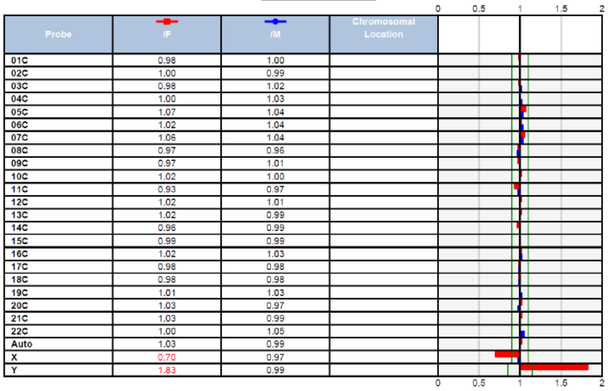

B

Normalized Ratios

Probe	/F	/M	Chromosomal Location
01C	1.01	1.02	
02C	1.02	1.03	
03C	0.92	0.96	
04C	0.98	1.04	
05C	0.97	1.02	
06C	1.06	1.02	
07C	0.99	1.01	
08C	0.92	1.02	
09C	0.96	1.00	
10C	1.00	0.98	
11C	1.01	1.08	
12C	1.04	1.10	
13C	1.01	1.05	
14C	1.04	1.00	
15C	1.07	1.01	
16C	0.96	0.96	
17C	1.03	1.05	
18C	0.98	1.00	
19C	1.01	1.06	
20C	0.98	1.04	
21C	1.08	1.02	
22C	0.95	1.00	
Auto	0.92	0.98	
X1;ptel	0.71	1.04	-
X2;pcen	0.66	0.96	-
X3;qcen	0.74	1.05	-
X4;qtel	0.76	1.05	-
Y1;ptel	1.58	1.09	-
Y2;pcen	3.05	1.08	-
Y3;qcen	1.46	0.96	-
Y4;qtel	2.28	1.06	-

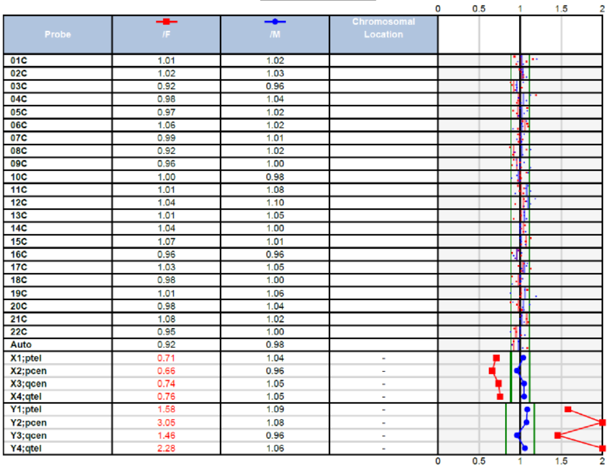

Normalized Ratios

Probe	/F	/M	Chromosomal Location
01C	1.01	1.02	
02C	1.02	1.03	
03C	0.92	0.96	
04C	0.98	1.04	
05C	0.97	1.02	
06C	1.06	1.02	
07C	0.99	1.01	
08C	0.92	1.02	
09C	0.96	1.00	
10C	1.00	0.98	
11C	1.01	1.08	
12C	1.04	1.10	
13C	1.01	1.05	
14C	1.04	1.00	
15C	1.07	1.01	
16C	0.96	0.96	
17C	1.03	1.05	
18C	0.98	1.00	
19C	1.01	1.06	
20C	0.98	1.04	
21C	1.08	1.02	
22C	0.95	1.00	
Auto	0.92	0.98	
X	0.72	1.04	
Y	1.93	1.07	

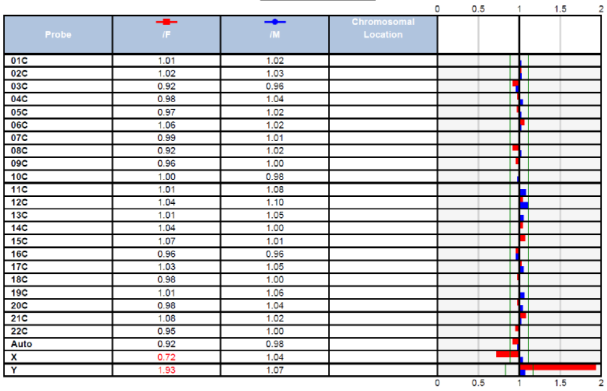

Supplementary Figure 5.

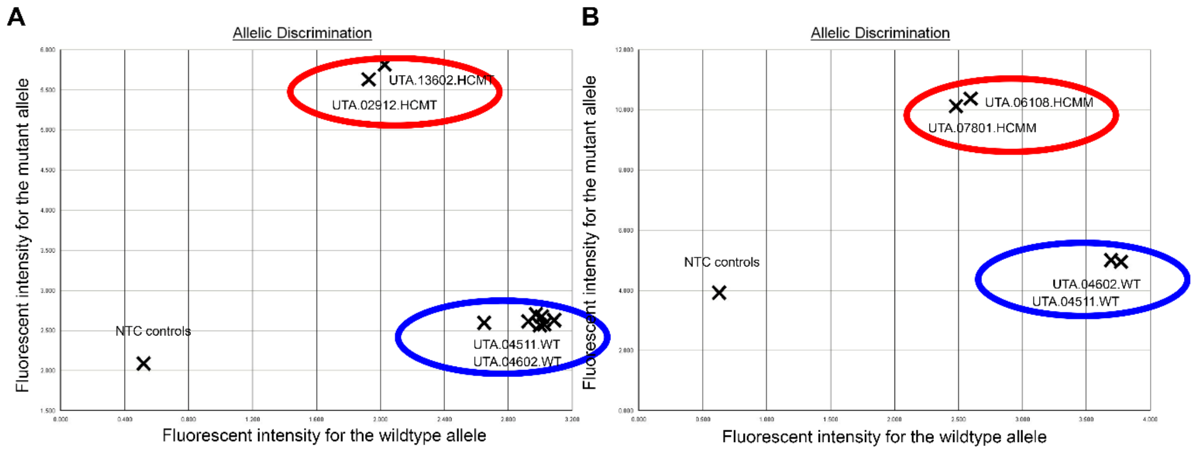

Supplementary Figure 6.

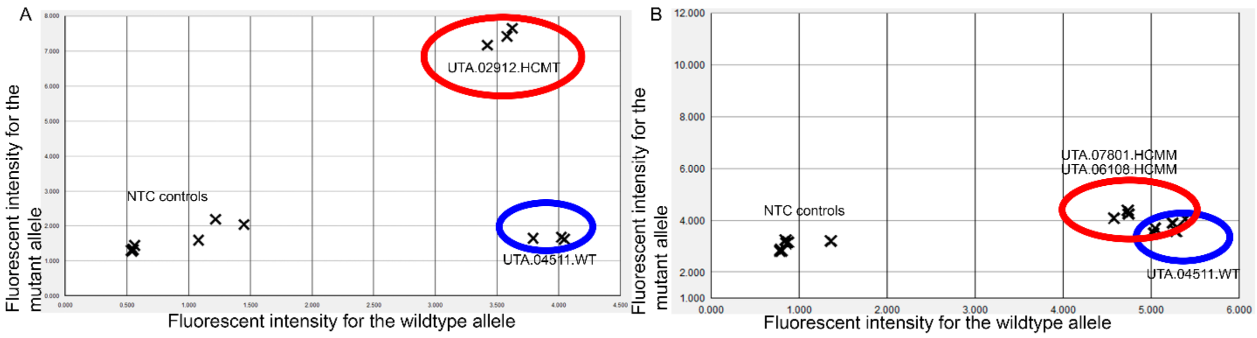

Supplementary Figure 7.

Mutation/application	Task	Dye	Sequence
**MYBPC-genotyping**	Forward primer		GCATTGAGAACATGGAGGACAAG
	Reverse primer		GCCCAGGGAAGGGAAACAAG
	Probe for the wild type allele	VIC	CCAACAACCT**G**CAGCAC
	Probe for the mutation allele	FAM	CCAACAACCT**A**CAGCAC
**MYBPC-mRNA**	Forward primer		GCATTGAGAACATGGAGGACAAG
	Reverse primer		TCAGTCACCCGGAGATCCT
	Probe for the wild type allele	VIC	CAACAACCT**G**CAGCACC
	Probe for the mutation allele	FAM	TCAACAACCT**A**CAGCACC
**TPM1Asp175Asn-genotyping and mRNA**	Forward primer		GGCCCGTAAGCTGGTCAT
	Reverse primer		CCGCTCCTCTGCACGTT
	Probe for the wild type allele	VIC	CATTGAGAGC**G**ACCTGG
	Probe for the mutation allele	FAM	ATTGAGAGC**A**ACCTGG

Supplementary Table1.
