# Supplementary material for: Mutation-Specific Phenotypes in hiPSC-Derived Cardiomyocytes Carrying Either Myosin-Binding Protein C Or α-Tropomyosin Mutation for Hypertrophic Cardiomyopathy
Source: Stem Cells Int. 2015 Dec 28;2016:1684792. doi: 10.1155/2016/1684792 (PMC4707351; doi:10.1155/2016/1684792)
Supplement: Supplementary file 1 — Supplementary Figure 1: Characterization of UTA.04511.WT cell line. Supplementary Figure 2: Characterization of UTA.02912.HCMT cell line. Supplementary Figure 3: Characterization of UTA.07801.HCMM cell line. Supplementary Figure 4: Characterization of UTA.06108.HCMM cell line. Supplementary Figure 5: Karyotype analyses of the cell lines A. UTA.04511.WT and B. UTA.02912.HCMT. Supplementary Figure 6: The presence of the TPM1-Asp175Asn and MYBPC3-Gln1061X mutation in the patient-specific hiPSC lines was confirmed by custom TaqMan SNP Genotyping Assays. Supplementary Figure 7: The mRNA expression of mutant and wildtype allelesin the hiPSC-derived CMs carrying TPM1-Asp175Asn or MYBPC3-Gln1061X mutation were assessed with the TaqMan SNP Genotyping Assays. Supplementary Table 1: Sequences of the primers and probes (Custom TaqMan SNP Genotyping Assays, Applied Biosystems, Life Technologies Ltd) used in the genotyping and mutant allele mRNA expression assays. [file 1684792.f1.zip › 1684792.f1/Supplementary material.docx]

**Supplementary Figure 1.** Characterization of UTA.04511.WT cell line. (a) The hiPSCs formed colonies expressing Nanog, OCT4, SOX2, SSEA4, TRA-1-60 and TRA-1-81. Scale bars 200 μm. (b) hiPSCs expressed endogenous SOX2 (151 bp), Nanog (287 bp), OCT4 (144 bp), c-MYC (328 bp) and Rex1 (306 bp). GAPDH (302 bp) was used as a housekeeping control. (c) The virally exported Sendai exogenes: exo-OCT4 (483 bp), exo-KLF4 (410 bp), exo-SOX2 (451 bp), exo-c-MYC (532 bp) were silenced in the hiPSCs. + indicates positive control. (d) Markers for all three germ layers were detected from the EBs. SOX17 (120 bp) was used as a marker for endoderm, SOX1 (166 bp), PAX6 (274 bp), NESTIN (208 bp) and Musashi (93 bp) were used as markers for ectoderm and VEGFR2/KDR (218 bp) as a marker for mesoderm.

**Supplementary Figure 2.** Characterization of UTA.02912.HCMT cell line. (a) The hiPSCs formed colonies expressing Nanog, OCT4 and SOX2. Scale bars 1.0mm. (b) hiPSCs expressed endogenous SOX2 (151 bp), Nanog (287 bp), OCT4 (144 bp), c-MYC (328 bp) and Rex1 (306 bp). GAPDH (302 bp) was used as a housekeeping control. (c) The virally exported Sendai exogenes: exo-OCT4 (483 bp), exo-KLF4 (410 bp), exo-SOX2 (451 bp), exo-c-MYC (532 bp) were silenced in the hiPSCs. + indicates positive control. (d) Markers for all three germ layers were detected from the EBs. SOX17 (166 bp) and AFP (209 bp) were used as markers for endoderm, PAX6 (275 bp) and NESTIN (208 bp) were used as markers for ectoderm and VEGFR2/KDR (218 bp) as a marker for mesoderm. GAPDH (302 bp) was used as a housekeeping control.

**Supplementary Figure 3.** Characterization of UTA.07801.HCMM cell line. (a) The hiPSCs formed colonies expressing Nanog, OCT4, SOX2, TRA-1-60 and TRA-1-81. Scale bars 200 μm. (b) The hiPSC line was karyotypically normal, 46 XY. (c) hiPSCs expressed endogenous Nanog (287 bp), OCT4 (144 bp), Rex1 (306 bp), SOX2 (151 bp) and c-MYC (328 bp). GAPDH (302 bp) was used as a housekeeping control. (d) Exogenes exo-OCT4 (225 bp), exo-KLF4 (250 bp), exo-SOX2 (200 bp) and exo-c-MYC (225 bp) were silenced in the hiPSCs. + indicates positive control. (e) Markers for all three germ layers were detected from the EBs. SOX17 (166 bp) and AFP (209 bp) were used as markers for endoderm, SOX1 (158 bp) and PAX6 (275 bp) were used as markers for ectoderm and VEGFR2/KDR (218 bp) as a marker for mesoderm. GAPDH (302 bp) was used as a housekeeping control.

**Supplementary Figure 4.** Characterization of UTA.06108.HCMM cell line. (a) The hiPSCs formed colonies expressing Nanog, SOX2, TRA-1-60, TRA-1-81 and OCT4. Scale bars 200 μm (OCT4: 100 μm). (b) hiPSCs expressed endogenous Nanog (287 bp), OCT4 (144 bp), Rex1 (306 bp), SOX2 (151 bp) and c-MYC (328 bp). GAPDH (302 bp) was used as a housekeeping control.(c) Exogenes exo-OCT4 (225 bp), exo-KLF4 (250 bp), exo-SOX2 (200 bp) and exo-c-MYC (275 bp) were silenced in the hiPSCs. + indicates positive control. (d) Markers for all three germ layers were detected from the EBs. SOX17 (166 bp) and AFP (209 bp) were used as markers for endoderm, SOX1 (158 bp) as a marker for ectoderm and VEGFR2/KDR (218 bp) as a marker for mesoderm. GAPDH (302 bp) was used as a housekeeping control. (e) The hiPSC line was karyotypically normal, 46 XY.

**Supplementary Figure 5.** Karyotype analyses of the cell lines A. UTA.04511.WT and B. UTA.02912.HCMT. Red and blue dots indicate chromosomal signal ratios of sample DNA against female (red) and male (blue) reference normal karyotype DNA, as detected by KaryoLiteTM BoBsTM assay. Signal from normal chromosomes against both male and female references should lie inside the reference area around value 1, whereas with an abnormal karyotype both signals lie outside the reference area. A female probe pattern is defined when X and Y probe ratios are included in the expected range for a female sample (red line/dots inside and blue line/dots outside the normal expected X/Y range); a male pattern is defined by a reverse pattern (blue line/dots inside and red line/dots outside the normal expected X/Y range). Each plot shows the signal of two technical replicates of the same sample. Both of the cell lines show normal karyotype.

**Supplementary Figure 6.** The presence of the TPM1-Asp175Asn and MYBPC3-Gln1061X mutation in the patient-specific hiPSC lines was confirmed by custom TaqMan SNP Genotyping Assays. The wildtype allele was detected by the probe labeled with VIC (fluorescent intensity on x-axis) and the mutated allele was detected by the probe labeled with FAM (the fluorescent intensity on y-axis). In the non-template controls (NTCs), water was used instead for the sample. (a) Presence of TPM1-Asp175Asn mutation in the UTA.13602.HCMT and UTA.02912.HCMT lines was observed due to the increased fluorescent intensity originating from the mutant allele specific probe on y-axis (marked with red circle). The fluorescent intensity of the mutant allele specific probe for the UTA.04511.WT and UTA.04602.WT is low and at the similar level with the NTC samples indicating that mutation is not detected in these control lines (blue circle). The wildtype allele was observed to be expressed at the similar manner in all the lines. (b) Presence of the MYBPC3-Gln1061X mutation in the UTA.06108.HCMM and UTA.07801.HCMM lines was observed due to the increased fluorescent intensity originating from the mutant allele specific probe on y-axis (marked with red circle). The fluorescent intensity of the mutant allele specific probe for the UTA.04511.WT and UTA.04602.WT is low and at the similar level with the NTC samples indicating that mutation is not detected in these control lines (blue circle). The wildtype allele was observed to be expressed at the similar manner in all the lines.

**Supplementary Figure 7.** The mRNA expression of mutant and wildtype allelesin the hiPSC-derived CMs carrying TPM1-Asp175Asn or MYBPC3-Gln1061X mutation were assessed with the TaqMan SNP Genotyping Assays. The wildtype allele was detected by the probe labeled with VIC (fluorescent intensity on x-axis) and the mutated allele was detected by the probe labeled with FAM (the fluorescent intensity on y-axis), similarly to the genotyping assay. In the non-template controls (NTCs), water was used instead for the sample. (a) CMs derived from UTA.02912.HCMT hiPSC line, carrying the TPM1-Asp175Asn mutation expressed both mutant and wildtype alleles on mRNA level (red circle), while CMs derived from UTA.04511.WT cell line expressed only wildtype allele (blue circle). (b) Clear difference in the expressions of wildtype and mutant alleles was not detected between HCM-CMs carrying the MYBPC3-Gln1061X mutation (UTA.07801.HCMM and UTA.06108.HCMM cell lines) and CMs derived from control hiPSCs (UTA.04511.WT cell line) indicating that the mutant allele expression is at low or the mRNA of the mutant allele is not present in the diseased lines.

**Supplementary Table 1.** Sequences of the primers and probes (Custom TaqMan SNP Genotyping Assays, Applied Biosystems, Life Technologies Ltd) used in the genotyping and mutant allele mRNA expression assays.
